# The role of metacognitive beliefs in impulse control disorders and addictive-like behaviors in Parkinson’s disease: an exploratory study

**DOI:** 10.3389/fpsyt.2026.1893510

**Published:** 2026-09-03

**Authors:** Sergio Triscari, Vittorio Lenzo, Alberto Sardella, Pasquale Caponnetto, Anna Galati, Patricia Lupica, Domenica Tasca, Stefano Muratore, Pietro Marano, Filippo Caraci, Maria C. Quattropani

**Affiliations:** 1 Department of Educational Sciences, Section of Psychology, University of Catania, Catania, Italy; 2 Neuropharmacology and Translational Neuroscience Unit, Oasi Research Institute – I.R.C.C.S, Troina, Italy; 3 Rehabilitation Psychology Unit, Department of Rehabilitation, Oasi Research Institute – I.R.C.C.S, Troina, Italy; 4 Neurorehabilitation Unit, Department of Rehabilitation, Oasi Research Institute – I.R.C.C.S, Troina, Italy; 5 Department of Drug and Health Sciences, University of Catania, Catania, Italy

**Keywords:** addiction, impulse control disorder, metacognition, metacognitive beliefs, Parkinson

## Abstract

**Background:**

Impulse control disorders (ICDs) are clinically relevant non-motor complications of Parkinson’s disease (PD). ICDs include gambling, hypersexuality, compulsive shopping, binge eating, hobbyism/punding, and dopamine dysregulation syndrome. Although dopaminergic replacement therapies are a key risk factor, only a subgroup of treated patients develop ICDs, potentially suggesting that neuropsychological differences may contribute to interindividual variability. Dysfunctional Metacognitive Beliefs (MBs) have recently been associated with addictive behaviors in the general population, but their role in neurologically driven addictive-like behaviors has not yet been examined. This exploratory study aimed to investigate whether dysfunctional MBs are associated with ICD symptom severity in PD patients after controlling for variables of dopaminergic exposure.

**Methods:**

38 PD patients (44.7% F, mean age 67.55) completed the Metacognitions Questionnaire-30 (MCQ-30) and the Questionnaire for Impulsive-Compulsive Disorders in Parkinson’s Disease-Rating Scale (QUIP-RS). One-sample t-tests were used to compare MCQ scores with normative values from the Italian general community sample. Two-tailed partial correlations tested associations between MCQ-30 scores and QUIP-RS outcomes, controlling for treatment duration, dopamine agonist exposure (LEDD-DA), and other dopaminergic exposures (i.e., total LEDD minus LEDD-DA). Additionally, independent samples t-tests were conducted to compare MCQ-30 scores between patients with and without ICDs. False discovery rate correction was applied.

**Results:**

Overall, 23.7% of patients exceeded the QUIP-ICD cut-off. Compared with normative data from the general community sample, PD patients showed a more dysfunctional metacognitive profile. After controlling for dopaminergic exposure, ICD symptom severity was associated with positive beliefs about worry, negative beliefs about uncontrollability and danger, reduced cognitive confidence, greater need to control thoughts, and lower cognitive self-consciousness. In group comparisons, patients exceeding the QUIP-ICD cut-off showed significantly higher MCQ-POS, MCQ-NEG, and MCQ-NC scores and lower MCQ-CSC scores than those below the cut-off.

**Discussion:**

These exploratory findings suggest that, after controlling for dopaminergic variables, dysfunctional MBs are associated with ICD symptom severity. In this context, MBs may represent one of the cognitive-affective correlates of ICD interindividual variability.

## 1 Introduction

### 1.1 Impulse control disorders (ICDs) in Parkinson’s disease: definition, incidence, and pathophysiology

#### 1.1.1 Clinical background and manifestations of ICDs

Parkinson’s disease (PD) is a chronic and progressive neurodegenerative condition essentially characterized by the degeneration of dopaminergic neurons in the pars compacta of the substantia nigra of the midbrain (1). This degeneration leads to a decrease in dopamine levels, which is a neurotransmitter essential for regulating movement as well as cognitive and emotional functions (1). Epidemiological data show that PD affects about 1% of the global population aged over 60, with a slightly higher prevalence in men than in women (2). Epidemiological projections also suggest that the absolute number of people living with PD may approximately double by 2040 (2). An interesting and often overlooked category of symptoms in PD is represented by Impulse Control Disorders (ICDs) (3). ICDs are behaviors marked by difficulty in resisting internal mental urges and images, which lead individuals to engage in compulsive actions to satisfy these urges. ICDs may include: (1) gambling, (2) compulsive shopping, (3) hypersexuality, (4) binge eating, and (5) problematic use of dopaminergic drugs, also known as Dopamine Dysregulation Syndrome (4). While ICDs are neurologically driven, their behavioral aspects configure them as addictive-like behaviors, adversely affecting the quality of life for patients and their caregivers.

#### 1.1.2 Dopaminergic mechanisms and interindividual variability in ICDs

The exact pathophysiology of ICDs in PD is not yet fully understood. Several studies support the crucial role of Dopamine Replacement Therapies (DRTs) in the development of these symptoms, due to the effect these therapies have on the brain’s reward systems (4–6). In patients with PD who are undergoing dopamine replacement therapies, the prevalence of ICDs varies significantly, ranging from 3.5% to 43% (4, 5, 7, 8). A wide range that highlights the heterogeneity of clinical cases among these patients. Indeed, Dopamine Agonists (DA) play a key role in controlling motor symptoms, as they provide compensatory dopaminergic stimulation to the nigrostriatal pathway, particularly through stimulation of D2 receptors (9). The problem arises when these drugs also exhibit high affinity for D3 receptors, which are relatively more represented at the level of ventral striatum and the nucleus accumbens, as well as in other limbic structures such as the thalamus, amygdala, and prefrontal areas (9). This results in an imbalance between reward processing and prefrontal inhibitory control. Among dopamine agonists, Pramipexole and Ropinirole are associated with the highest risk of ICDs, respectively 17.7% and 15.5% (10). In contrast, the risk of ICD with Rotigotine is lower, ranging from 2% to 9% (10), due to its transdermal administration. Even Levodopa alone, particularly at high doses and during long-term treatment, has also been associated with an increased risk of ICDs (10). Despite these considerations, the clinical evidence that approximately 6.9-17.5% of PD patients meet the criteria for ICDs without any significant association with drug therapy (11, 12), as well as the fact that not all patients treated with dopamine replacement therapies develop ICDs (10), leads us to hypothesize that drug therapy alone is not sufficient to explain this phenomenon in its entirety. In sum, the critical issue is no longer whether dopamine replacement therapies affect ICDs, this is well established in the literature, but why only some patients exhibit significant behavioral dysregulation in response to a shared dopaminergic pharmacological stressor.

### 1.2 The possible role of metacognitive beliefs on ICDs

Building on the previous question, we hypothesized that the association between dopamine replacement therapies and symptoms of ICDs may vary depending on specific neuropsychological differences. In this study, we specifically explore the construct of metacognitive beliefs (MB) as one of the potential neuropsychological correlates of ICDs. The term Metacognition (MC) refers to the set of cognitive processes involved in the evaluation, monitoring, and control of thinking (13, 14). More recently, thanks to the work of Wells and colleagues (15), metacognition has come to be understood not merely as the ability to monitor one’s own thinking, but also as a set of Metacognitive Beliefs (MBs) that regulate how individuals respond to the content of their internal thoughts (15, 16). From this perspective, two individuals may experience similar intrusive thoughts or impulses. However, differences in the clinical expression of these experiences may be associated not only with the impulses themselves, but also with their metacognitive response to that internal activation. Specifically, the authors developed a model known as the Self-Regulatory Executive Function (S-REF), whose metacognitive architecture was later further specified through the Metacognitive Control System (MCS) (17, 18). MCS is responsible for monitoring and regulating cognitive processing. In this scenario, MBs constitute an important form of declarative information within this system and concern beliefs about the meaning, usefulness, controllability, and consequences of cognitive activity (18). MBs focus specifically on five key areas: positive beliefs about worry, negative beliefs about uncontrollability and danger, cognitive confidence, need to control thoughts, and cognitive self-consciousness (16). These dimensions represent aspects of declarative metacognitive knowledge implicated in the regulation of cognitive processing within the S-REF/MCS framework (18). Individuals typically activate these beliefs in an effort to manage or reduce their distress. However, these beliefs may promote the selection of regulatory strategies that, rather than alleviating distress, can increase negative affectivity and perpetuate the cycle of behavioral dysregulation. Indeed, dysfunctional MBs may contribute to a cascade of maladaptive metacognitive regulation within the broader MCS, which may in turn sustain maladaptive and perseverative patterns of cognitive processing. Evidence supports the transdiagnostic relevance of these MBs: a meta-analytic review found dysfunctional metacognitive beliefs across a broad range of psychopathologies, with particularly robust effects for negative beliefs concerning uncontrollability and danger and beliefs about the need to control thoughts (19). Previous work has linked dysfunctional metacognitive beliefs to addictive bahaviors of the general population (20), supporting the view that the way MBs influence and regulate craving-related or intrusive mental contents may contribute to maladaptive bahavioral outcomes (20). However, this framework has mainly been investigated in non-neurological and non-iatrogenic forms of addictive-like bahaviors (21), whereas little is known about its applicability to ICD symptoms occurring in PD patients treated with dopamine replacement therapies. ICD symptoms in PD and related conditions emerge within a complex interaction. For this reason, we selected MBs as the principal psychological construct of interest in the present study. MBs may represent a possible relevant psychological cognitive-affective correlate of ICD symptoms. Given this background, the present study adopted an exploratory correlational design. We aimed to examine whether MBs were associated with the severity of ICD symptoms in patients receiving dopamine replacement therapies, and whether these associations remained statistically significant after controlling for key dopaminergic treatment-related variables. Our primary hypothesis was that more dysfunctional MBs were associated with greater symptoms of ICD, even when controlling for dopaminergic exposure variables.

## 2 Methods

### 2.1 Participants

This study employed a cross-sectional design. 38 patients with idiopathic Parkinson’s disease were recruited from the Oasi Research Institute - I.R.C.C.S. All participants were hospitalized at the time of assessment and underwent a comprehensive clinical and neuropsychological evaluation during the ON phase, including assessment of exposure to dopaminergic medications. All patients had a confirmed diagnosis of idiopathic PD, established by specialist neurological evaluation and supported by standardized clinical and instrumental assessments, including the Movement Disorder Society-Unified Parkinson’s Disease Rating Scale (MDS-UPDRS) (22) and dopamine transporter imaging with DaTSCAN. Eligibility criteria included a Hoehn and Yahr (H. & Y.) (23) stage ≤ 3, absence of severe cognitive impairment, operationalized as a Montreal Cognitive Assessment (MoCA) (24, 25) score ≤ 12, and absence of severe psychiatric disorders that could interfere with the validity or reliability of clinical and neuropsychological assessment. The study was conducted in accordance with the ethical principles of the 1964 Declaration of Helsinki and its subsequent amendments. Ethical approval was obtained from the Internal Ethics Review Board (IERB) for Psychological Research at the University of Catania (Prot. No. Ierb-Edunict-2025.09.26/01). All participants provided written informed consent before taking part in the study. Privacy and personal data were protected in accordance with the European Union General Data Protection Regulation (EU GDPR 2016/679).

### 2.2 Measures

Sociodemographic and clinical data, including age, gender, educational level, employment status, marital status, and treatment duration, were collected during recruitment. Cognitive screening included the MoCA, used to identify severe cognitive impairment according to the exclusion criteria, and the Frontal Assessment Battery (FAB) (26), used as a brief measure of frontal-executive functioning. General psychopathological symptoms were screened using the DSM-5-TR Level 1 Cross-Cutting Symptom Measure (27), a transdiagnostic tool assessing psychiatric symptom domains. Consistent with its intended use, scores at or above the screening threshold, commonly ≥2 for most domains, were considered indicators of symptoms requiring additional clinical evaluation. Screening results were integrated with the clinical judgment of the psychologists involved in the patients’ care. This combined screening and clinical evaluation procedure was used during the eligibility phase to reduce heterogeneity in psychiatric symptom burden at baseline and to ensure that included patients did not have severe psychiatric comorbidities. After completion of the screening and clinical characterization procedures, the main variables of interest were assessed through the following standardized measures:

- Levodopa Equivalent Daily Dose (LEDD), Dopamine Agonist Levodopa Equivalent Daily Dose (LEDD-DA), and Treatment Duration. Dopaminergic treatment exposure was quantified using the LEDD, expressed in mg/day. LEDD provides a standardized estimate of overall dopaminergic medication burden by converting different antiparkinsonian drugs into a common levodopa-equivalent metric, thereby accounting for differences in pharmacological potency, route of administration, and drug-specific conversion factors (28, 29). For each participant, current dopaminergic medications were extracted from the clinical pharmacological record at the time of assessment. Daily doses were calculated and converted into levodopa-equivalent values according to established conversion procedures. Specifically, levodopa was converted using a 1:1 ratio, whereas dopamine agonists were converted using potency-based factors: pramipexole daily dose x 100, ropinirole daily dose x 20, and rotigotine daily dose x 30 (29). Total LEDD was obtained by summing all levodopa-equivalent components. In addition, dopamine agonist-specific exposure was calculated separately as LEDD-DA, including only the levodopa-equivalent contribution of pramipexole, ropinirole, and rotigotine. Thus, total LEDD indexed overall dopaminergic medication burden, whereas LEDD-DA indexed dopamine agonist exposure, given its established relevance to ICD symptoms in PD (9, 10). Treatment duration was also extracted from the clinical pharmacological record and expressed in years. In the present study, treatment duration was used as an additional index of dopaminergic exposure over time, complementing current dose-based measures such as total LEDD and LEDD-DA. This variable was included as a covariate in the partial correlation analyses to account, at least partially, for the temporal dimension of dopaminergic treatment exposure
- The Questionnaire for Impulsive-Compulsive Disorders in Parkinson’s Disease -Rating Scale (QUIP-RS) (30, 31). The QUIP-RS is a PD specific self-report or clinician-administered rating scale designed to quantify the frequency and severity of ICD symptoms over the previous four weeks. It consists of four core questions, rated on a 5-point Likert scale from 0 = “Never” to 4 = “Very often”, applied to each bahavioral domain. Examples of questions include: “How much do you think about the following behaviors?” or “Do you have urges or desires for the following behaviors that you feel are excessive or cause you distress?”. Higher scores indicate greater frequency and severity of ICD symptoms. In the present study, the QUIP-RS was clinician-administered as a standardized semi-structured interview. All patients were assessed using the predefined QUIP-RS domains, items, time frame, and scoring anchors, while being allowed to provide free clinical descriptions to clarify symptom frequency and severity. These responses were used only to guide scoring according to the established QUIP-RS criteria. QUIP-RS assessors were blinded to participants’ MCQ-30 scores during administration and scoring. Domain-specific scores were included in the analyses, together with an ICD composite score (QUIP-ICD) comprising gambling, hypersexuality, compulsive shopping, and binge eating. Moreover, we have also included an overall QUIP-RS score that includes the QUIP-ICD score plus hobbyism/punding score and dopamine dysregulation syndrome score. To identify clinically significant ICD behaviors, we utilized cut-offs recognized in the literature: gambling ≥ 6, hypersexuality ≥ 8, compulsive shopping ≥ 8, compulsive eating ≥ 7, hobbyism/punding ≥ 7, and QUIP-ICD score ≥ 10 (30). Since no validated cut-off scores have been established for dopamine dysregulation syndrome or for the QUIP-RS total score, these conditions were interpreted qualitatively. The QUIP-RS was used to assess ICD-related symptom frequency and severity, and established cut-offs were used to identify clinically relevant symptom levels rather than to establish formal ICD diagnoses. In our sample, the Italian version of QUIP-RS (31) showed excellent internal consistency with Cronbach’s alpha values of 0.85.
- The Metacognitions Questionnaire (MCQ-30) (16, 32, 33). The MCQ-30 is a 30-item self-report measure rated on a 4-point Likert scale, from 1 = “Do not agree” to 4 = “Agree very much”. The questionnaire assesses five domains of metacognition: positive beliefs about worry (POS), negative beliefs about uncontrollability and danger (NEG), lack of cognitive confidence (CC), need to control thoughts (NC), and cognitive self-consciousness (CSC). Examples of items include: “Worrying helps me to avoid problems in the future” (MCQ-POS) or “My worrying could make me go mad” (MCQ-NEG) or “I have little confidence in my memory for actions” (MCQ-CC) or “I will be punished for not controlling certain thoughts” (MCQ-NC) or “I monitor my thoughts” (MCQ-CSC). Total and subscale scores were included in the analysis to explore both global and domain-specific associations. In our sample, the Italian version of MCQ-30 (32, 33) showed excellent internal consistency with Cronbach’s alpha values of 0.87 for MCQ-POS, 0.93 for MCQ-NEG, 0.95 for MCQ-CC, 0.81 for MCQ-NC, 0.96 for MCQ-CSC, and 0.96 for MCQ total (MCQ-TOT).

### 2.3 Statistical analysis

Statistical analyses were performed using IBM SPSS Statistics, version 32 (34). Descriptive statistics were calculated for sociodemographic, clinical, pharmacological, and psychometric variables. Continuous variables were summarized using means, standard deviations, medians, and ranges, whereas categorical variables were reported as frequencies and percentages. The proportion of patients exceeding established QUIP-RS cut-off scores was calculated to describe the prevalence of clinically relevant ICD symptoms in the sample. Internal consistency of the main psychometric instruments was evaluated using Cronbach’s alpha. To characterize the metacognitive profile of the sample, one-sample t-tests were used to compare MCQ-30 scores with normative values from the general community sample included in the Italian validation study (32). One-way ANOVAs were used to compare continuous demographic, clinical, and cognitive variables, including age, disease-onset variables, MoCA corrected score, FAB corrected score, and pharmacological exposure. Gender was treated as a categorical variable and compared between ICD-positive and ICD-negative patients using Fisher’s exact test. These preliminary analyses were intended to describe group comparability and to evaluate whether major demographic or clinical differences were evident between ICD-positive and ICD-negative patients. Partial correlation analyses were then conducted to examine associations between MCQ-30 total and subscale scores and QUIP-RS domain and composite scores. In these analyses, exposure to dopaminergic medication was controlled by including treatment duration, dopamine agonist-specific exposure, indexed by LEDD-DA, and non-dopamine agonist dopaminergic exposure, computed as total LEDD minus LEDD-DA, as covariates. This approach allowed us to examine whether associations between metacognitive beliefs and ICD symptoms remained significant after accounting for variables of pharmacological exposure. Pearson correlations were also used to examine associations between dopaminergic exposure indices and QUIP-RS domain and composite scores. Complementary exploratory partial correlations then assessed whether any observed associations persisted after controlling for MCQ-30 total score. To address the issue of statistical power in the context of the available clinical sample, a *post hoc* sensitivity power analysis for partial correlations was conducted. To account for multiple testing, p-values from partial correlation analyses were adjusted using the Benjamini-Hochberg false discovery rate procedure (35, 36). Correction was applied separately to the primary correlation family involving QUIP-ICD and QUIP-RS composite scores with MCQ-30 scores, and to the secondary exploratory domain analyses involving every single ICD score with MCQ-30 scores. For p-values reported by SPSS as p <.001, a conservative value of p = .001 was used for FDR computation. In addition, exploratory group comparisons were conducted between patients who exceeded the QUIP-ICD cutoff and those who did not. Independent-samples t-tests were used to compare MCQ-30 total and subscale scores between groups. Effect sizes were reported using Cohen’s d. All analyses were two-tailed, and statistical significance was set at p <.05. Statistical assumptions were assessed using Shapiro-Wilk tests, skewness and kurtosis indices, Q-Q plots, boxplots, Levene’s test, and regression diagnostics for potentially influential observations. Given the exploratory nature of the study and the limited sample size, findings were interpreted primarily in terms of effect size, expected direction, and consistency of associations.

## 3 Results

### 3.1 Characteristics of the participants

**Table 1**. shows the demographic and clinical characteristics of the participants. A total of 43 patients with idiopathic Parkinson’s disease (PD) were initially screened. Of these, five were excluded because they did not meet the inclusion criteria for idiopathic PD, resulting in a final sample of 38. Participants had a mean age of 67.55 years (SD = 7.92; median = 67.50). The sample had a balanced gender distribution (F = 44.7% vs M = 55.3%), with a slight predominance of men, consistent with the higher incidence of PD among men (2). H. & Y. disease severity indicated a mild stage of disease progression in the sample (mean = 2.82; SD = 0.39; median = 3.00; mode = 3.00). The mean treatment duration was 8.68 years (SD = 5.82; median = 8.00). At screening, the patient group showed global cognitive functioning below the normal limits (MoCA mean score = 20.62; SD = 4.57; median = 21.40) with peculiar deficits affecting frontal functioning (FAB mean score = 12.06; SD = 3.02; median = 12.04) (26, 37). Most participants were married (n=24, 63.2%), followed by divorced (n=6, 15.8%), widowed (n=5, 13.2%), and single participants (n=3, 7.9%). Educational level was generally low to moderate: primary school (n=9, 23.7%), lower secondary school (n=15, 39.5%), upper secondary school diploma (n=8, 21.1%), university degree (n=5, 13.2%), and postgraduate specialization or PhD (n=1, 2.6%). With respect to current employment status, most participants were retired (n = 27, 71.1%). The remaining participants were housewives (n=4, 10.5%), craftsmen (n=2, 5.3%), farmers (n = 2, 5.3%), managers (n=2, 5.3%), or unemployed (n=1, 2.6%). Among participants retired, the most frequently reported occupational categories were skilled worker (n=5, 17.9%), and public employee (n=5, 17.9%), followed by housewife (n=4, 14.3%), craftsman (n=3, 10.7%), farmer (n=3, 10.7%), manager (n=3, 10.7%), dealer (n=3, 10.7%), and military police service (n=1, 3.6%).

**Table 1:** Demographic and clinical characteristics of participants (N = 38).

Characteristic	Statistics/category	Value
Age, years	Mean (SD); median	67.55 (7.92); 67.50
H. & Y.	Mean (SD); median; mode	2.82 (0.39); 3; 3
Treatment duration, years	Mean (SD); median	8.68 (5.82); 8.00
Global cognitive functioning	MoCA score, mean (SD); median	20.62 (4.57); 21.40
Frontal functioning	FAB score, mean (SD); median	12.06 (3.02); 12.04
Sex	Female	17 (44.7%)
	Male	21 (55.3%)
Marital status	Married	24 (63.2%)
	Divorced	6 (15.8%)
	Widowed	5 (13.2%)
	Single	3 (7.9%)
Educational level	Primary school	9 (23.7%)
	Lower secondary school	15 (39.5%)
	Upper secondary school diploma	8 (21.1%)
	University degree	5 (13.2%)
	Postgraduate specialization or PhD	1 (2.6%)
Employment status	Retired	27 (71.1%)
	Housewife	4 (10.5%)
	Craftsman	2 (5.3%)
	Farmer	2 (5.3%)
	Manager	2 (5.3%)
	Unemployed	1 (2.6%)
Previous occupation if retired	Skilled worker	5 (17.9%)
	Public employee	5 (17.9%)
	Housewife	4 (14.3%)
	Craftsman	3 (10.7%)
	Farmer	3 (10.7%)
	Manager	3 (10.7%)
	Dealer	3 (10.7%)
	Military/Police service	1 (3.6%)

### 3.2 Prevalence and severity of impulse control disorders symptoms

The QUIP-ICD composite score, calculated as the sum of gambling, hypersexuality, compulsive shopping, and binge eating, showed a mean of 5.08 (SD = 5.97; median = 2.50). Based on the established QUIP-ICD cut-off score of ≥ 10 (30, 31), 23.7% of participants exceeded the threshold for clinically relevant ICD symptoms (n = 9, 23.7%; 95% CI: 13.0-39.2). The overall QUIP-RS score, which additionally included hobbyism/punding and dopamine dysregulation syndrome, showed a mean of 8.71 (SD = 9.25; median = 5.50). At the level of single domains, scores above the cut-off were observed for gambling (n=3, 7.9%), hypersexuality (n=2, 5.3%), Hobbyism/punding (n = 5, 13.2%), and binge eating (n=3, 7.9%). No participant exceeded the established cut-off for compulsive shopping. Dopamine dysregulation syndrome was not classified using a cut-off because no validated threshold is currently available; therefore, this domain was interpreted only qualitatively. In this domain, 13.2% of participants reported non-zero symptoms (n=5). To further characterize the distribution of clinically relevant ICDs, participants were classified according to the number of QUIP domains exceeding validated cut-offs. Overall, 26.3% of participants exhibited at least one domain above the cut-off (n = 10), while 1 participant met the criteria for multi-ICD symptoms, defined as having at least two domains above the cut-off (n=1, 2.6%). The case involved gambling, hypersexuality, and binge eating. In terms of dimensional severity, the highest mean domain score was observed for hobbyism/punding (M = 2.87, SD = 3.77; median = 1.00; range = 0–17), followed by hypersexuality (M = 1.82, SD = 2.99; median = 0.00; range = 0-10), binge eating (M = 1.79, SD = 2.56; median = 0.00; range = 0-9), medication-related symptoms (M = 0.76, SD = 2.34; median = 0.00; range = 0-10), gambling (M = 0.74, SD = 1.96; median = 0.00; range = 0-8), and compulsive shopping (M = 0.74, SD = 1.80; median = 0.00; range = 0-7). Pearson correlations showed no significant associations between dopaminergic pharmacological exposure indices and the main ICD composite measures, as total LEDD, LEDD-DA, and non-DA LEDD were not significantly correlated with either QUIP-ICD or QUIP-RS total scores. However, at the domain level, LEDD-DA was positively associated with binge eating symptoms, r = .387, p = .016. Treatment duration was positively associated with QUIP-RS total score, r = .326, p = .045, and dopamine dysregulation symptoms, r = .421, p = .009; its association with gambling symptoms was in the same direction but did not reach significance, r = .318, p = .052. In complementary partial correlations controlling for MCQ-TOT, the associations of LEDD-DA with binge eating symptoms, r = .414, p = .011, and treatment duration with dopamine dysregulation symptoms, r = .367, p = .025, remained positively associated and significant, although the latter was attenuated. Conversely, the association between treatment duration and QUIP-RS total score was attenuated and no longer significant, r = .218, p = .195.

### 3.3 Metacognitive profile of Parkinson’s patients

To descriptively characterize the metacognitive profile of the sample, one-sample t-tests were conducted comparing MCQ-30 scores with Italian normative values from the general community populations (32). Overall, patients with PD showed a more dysfunctional metacognitive profile than the general community sample. The MCQ-30 total score was significantly higher than the Italian general community normative mean, M = 70.76, SD = 9.01 vs. 60.34, t(37) = 7.13, p <.001, mean difference = 10.42, 95% CI [7.46, 13.39], with a large effect size, Cohen’s d = 1.16. At the subscale level, patients reported significantly higher negative beliefs about uncontrollability and danger MCQ-NEG, M = 16.95, SD = 5.17 vs. 11.55, t(37) = 6.43, p <.001, d = 1.04; lower cognitive confidence, reflected by higher MCQ-CC scores, M = 15.21, SD = 5.08 vs. 9.94, t(37) = 6.39, p <.001, d = 1.04; and a greater need to control thoughts, M = 14.79, SD = 3.53 vs. 11.71, t(37) = 5.38, p <.001, d = 0.87. Conversely, patients scored lower than normative values on positive beliefs about worry MCQ-POS, M = 9.42, SD = 2.66 vs. 10.49, t(37) = −2.48, p = .018, d = −0.40, and cognitive self-consciousness MCQ-CSC, M = 14.39, SD = 6.30 vs. 16.65, t(37) = −2.21, p = .034, d = −0.36. However, comparisons with general community normative data were intended only as descriptive context and should be interpreted cautiously because the samples were not matched for neurological status.

### 3.4 Association between metacognitive beliefs and ICD

Before examining the association between MB and ICD symptoms severity, preliminary group comparisons were conducted to evaluate whether patients exceeding the QUIP-ICD cut-off differed from those below the cut-off on major demographic, clinical, and cognitive variables. No significant between-group difference emerged for the disease-onset variable, F(1,36) = 0.20, p = .660, η² = .005. Age also did not differ significantly between groups, although the effect was non negligible and should be interpreted cautiously given the limited sample size, F(1,36) = 3.18, p = .083, η² = .081. Gender distribution did not significantly differ between ICD-positive and ICD-negative patients, although the ICD-positive group showed a higher proportion of men (7/9, 77.8% vs. 14/29, 48.3%; Fisher’s exact test, p = .148). Similarly, no significant group differences were observed in global cognitive functioning, as indexed by the MoCA corrected score, F(1,36) = 1.23, p = .274, η² = .033, or in frontal-executive functioning, as indexed by the FAB corrected score, F(1,36) = 0.31, p = .580, η² = .009. No significant between-group differences were also found for H. & Y. stage, F(1,36) = 0.17, p = .679, η² = .005. Thus, within a sample already characterized by reduced cognitive and frontal-executive performance, cognitive status did not appear to distinguish patients with clinically relevant ICD symptoms from those without ICD symptoms. These preliminary analyses suggest that subsequent differences in metacognitive beliefs are unlikely to be simply explained by major demographic imbalance or by gross differences in global cognition or executive functioning.

Pharmacological exposure was also compared between patients exceeding the QUIP-ICD cut-off and those below the cut-off. No significant between-group differences emerged for total LEDD, F(1,36) = 0.07, p = .794, η² = .002, LEDD-DA, F(1,36) = 0.004, p = .948, η² <.001, or non-dopamine agonist dopaminergic exposure non-DA LEDD, F(1,36) = 0.08, p = .785, η² = .002. In the total sample, mean total LEDD was 704.74 mg/day (SD = 482.90; median = 607.50; range = 100–2220), mean LEDD-DA was 54.87 mg/day (SD = 79.04; median = 0.00; range = 0–300), and mean non-DA LEDD was 649.87 mg/day (SD = 481.07; median = 500.00; range = 100–2220). Thus, patients with and without clinically relevant ICD symptoms did not differ in overall dopaminergic burden or dopamine agonist-specific exposure. Nevertheless, given the small sample size, all of these comparisons should be interpreted descriptively.

Regarding psychotropic medication use, exposure was first examined globally as the presence of at least one psychotropic or centrally acting neuropsychiatric medication. Overall, 29 participants (76.3%) were receiving at least one such medication, whereas 9 participants (23.7%) were not receiving any psychotropic medication. At the level of specific pharmacological classes, benzodiazepines/anxiolytics were the most frequent treatment (n = 18, 47.4%), followed by antidepressants (n = 14, 36.8%), antipsychotics (n = 9, 23.7%), antiepileptic/mood-stabilizing agents (n = 3, 7.9%), and cognitive-enhancing drugs (n = 2, 5.3%). Twelve participants (31.6%) were receiving two psychotropic medication classes. Psychotropic exposure did not differ between ICD-positive and ICD-negative participants when considered globally as exposure to at least one psychotropic medication (66.7% vs. 79.3%, Fisher’s exact test, p = .655). Similarly, no significant group differences were observed for the main pharmacological classes, including antidepressants (22.2% vs. 41.4%, p = .438), benzodiazepines/anxiolytics (33.3% vs. 51.7%, p = .454), and antipsychotics (22.2% vs. 24.1%, p = 1.000). At the dimensional level, exploratory Spearman’s correlations showed that QUIP-ICD severity was not associated with the number of psychotropic medication classes, ρ = −.051, p = .760, or with total available psychotropic burden indexed by Defined Daily Dose (DDD) ratios, ρ = .049, p = .768. No significant associations were also found for class-specific dose indices, including benzodiazepine diazepam-equivalent dose and antidepressant, antipsychotic, antiepileptic/mood-stabilizing, or cognitive-enhancing DDD ratios, all ps >.29. These exploratory findings suggest that psychotropic medication exposure and burden were not related to greater ICD symptom severity in this sample.

Partial correlation analyses were then performed to examine whether metacognitive beliefs were associated with ICD symptoms after controlling for dopaminergic treatment exposure. Specifically, treatment duration, dopamine agonist-specific exposure, indexed by LEDD-DA, and non-dopamine agonist dopaminergic exposure, computed as total LEDD minus LEDD-DA, were entered as covariates. To account for multiple testing, p-values were adjusted using the Benjamini-Hochberg false discovery rate procedure. Correction was applied separately to the primary and secondary correlation family. The primary family of tests was defined *a priori* as associations between MCQ-30 scores and the two composite ICD outcomes. Domain-level QUIP-RS analyses were considered descriptively and exploratory.

**Figure 1:**
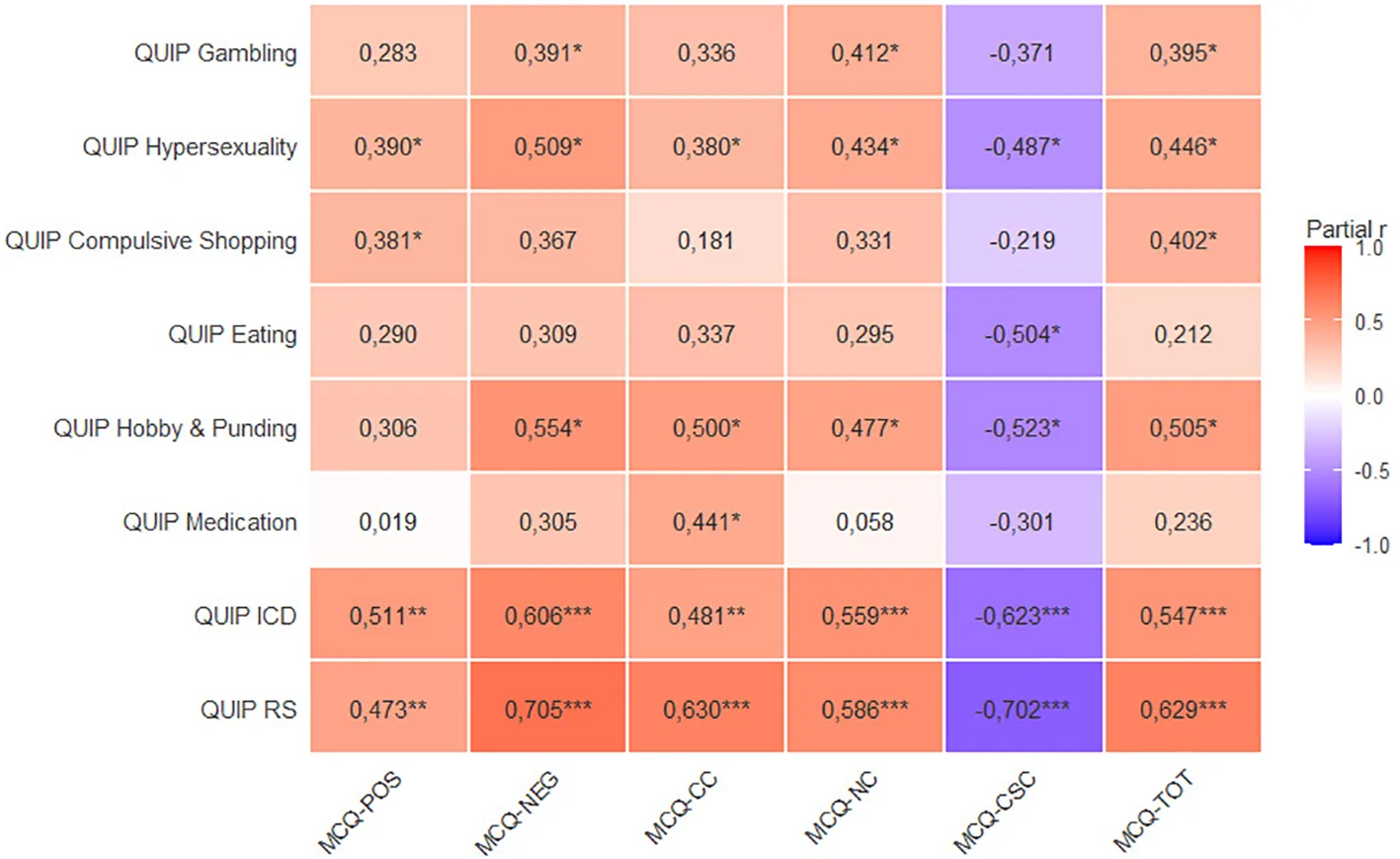
Heatmap of partial correlations between metacognitive beliefs and impulse control disorder symptoms in PD. Statistical significance is based on FDR-adjusted p-values: * p < .05, ** p < .01, *** p ≤ .001.

**Figure 1** summarized the partial correlation results. After controlling for pharmacological variables, the QUIP-ICD composite score showed significant associations with all MCQ-30 domains, and all associations remained significant after FDR correction. Specifically, Higher ICD severity was positively associated with positive beliefs about worry (MCQ-POS), r = .511, p = .002, pFDR = .002; negative beliefs about uncontrollability and danger (MCQ-NEG), r = .606, p <.001, pFDR = .001; reduced cognitive confidence (MCQ-CC), r = .481, p = .003, pFDR = .003; greater need to control thoughts (MCQ-NC), r = .559, p <.001, pFDR = .001; and higher MCQ total score, r = .547, p <.001, pFDR = .001. Conversely, ICD severity was negatively associated with cognitive self-consciousness (MCQ-CSC), r = -.623, p <.001, pFDR = .001, indicating that patients with more severe ICD symptoms reported lower levels of reflective monitoring of their own thoughts. A similar pattern was observed for the QUIP-RS total score, which included ICD symptoms together with hobbyism/punding and dopamine dysregulation syndrome. The QUIP-RS total score was positively associated with MCQ-POS, r = .473, p = .004, pFDR = .004; MCQ-NEG, r = .705, p <.001, pFDR = .001; MCQ-CC, r = .630, p <.001, pFDR = .001; MCQ-NC, r = .586, p <.001, pFDR = .001; and MCQ total score, r = .629, p <.001, pFDR = .001. QUIP-RS total score was also negatively associated with MCQ-CSC, r = -.702, p <.001, pFDR = .001.

A *post hoc* sensitivity power analysis was conducted using G*Power 3.1.9.7 to contextualize the statistical sensitivity of the study. With N = 38, three covariates partialled out, and a two-tailed α level of.05, statistical power was limited for a conventional medium partial correlation, r = .30, 1 - β = .43, but sufficiently for a conventional large partial correlation, r = .50, 1 - β = .88. Therefore, the study was underpowered to detect conventional medium associations but powered to detect conventional large associations. This is consistent with the magnitude of the observed primary partial correlations.

At the level of individual QUIP-RS domains, partial correlations were considered descriptive and were not interpreted as domain-specific evidence. After FDR correction, gambling scores showed significant positive associations with MCQ-NEG, r = .391, p = .020, pFDR = .045; MCQ-NC, r = .412, p = .014, pFDR = .042; and MCQ total score, r = .395, p = .019, pFDR = .045. Hypersexuality scores showed significant positive associations with MCQ-POS, r = .390, p = .020, pFDR = .045; MCQ-NEG, r = .509, p = .002, pFDR = .012; MCQ-CC, r = .380, p = .024, pFDR = .048; MCQ-NC, r = .434, p = .009, pFDR = .029; and MCQ total score, r = .446, p = .007, pFDR = .028, as well as a significant negative association with MCQ-CSC, r = -.487, p = .003, pFDR = .015. Compulsive shopping scores showed significant positive associations with MCQ-POS, r = .381, p = .024, pFDR = .048, and MCQ total score, r = .402, p = .017, pFDR = .045. Binge eating scores showed a significant negative association only with MCQ-CSC, r = -.504, p = .002, pFDR = .012. Hobbyism/punding scores showed significant positive associations with MCQ-NEG, r = .554, p <.001, pFDR = .012; MCQ-CC, r = .500, p = .002, pFDR = .012; MCQ-NC, r = .477, p = .004, pFDR = .018; and MCQ total score, r = .505, p = .002, pFDR = .012, as well as a significant negative association with MCQ-CSC, r = -.523, p = .001, pFDR = .012. Finally, dopamine dysregulation syndrome scores showed a significant positive association only with MCQ-CC, r = .441, p = .008, pFDR = .029.

Independent samples t-tests were then conducted to compare MCQ-30 scores between patients above and below the QUIP-ICD cut-off. After FDR correction across the six MCQ-30 comparisons, ICD-positive patients showed significantly higher scores on MCQ-POS, 11.22 ± 1.72 vs. 8.86 ± 2.67, t(21.12) = 3.12, p = .005, pFDR = .010, d = 0.95; MCQ-NEG, 20.89 ± 2.57 vs. 15.72 ± 5.19, t(28.15) = 4.01, p <.001, pFDR = .003, d = 1.09; and MCQ-NC, 17.22 ± 3.07 vs. 14.03 ± 3.35, t(36) = 2.54, p = .016, pFDR = .024, d = 0.97. ICD-positive patients also showed significantly lower MCQ-CSC scores, 9.22 ± 3.15 vs. 16.00 ± 6.19, t(27.37) = −4.35, p <.001, pFDR = .003, d = −1.20. The between-group difference in MCQ total score did not survive FDR correction, although the effect size was medium-to-large, 75.89 ± 7.57 vs. 69.17 ± 8.94, t(36) = 2.03, p = .049, pFDR = .059, d = 0.78. No statistically significant difference emerged for MCQ-CC, 17.33 ± 5.68 vs. 14.55 ± 4.80, t(36) = 1.46, p = .154, pFDR = .154, d = 0.56.

Assumption checks identified no issues that materially affected the analyses; Welch’s correction was applied where appropriate, and sensitivity analyses confirmed that the main findings were not driven by influential observations.

## 4 Discussion

To the best of our knowledge, this is the first study to investigate the potential link between ICD symptoms and Metacognitive Beliefs in PD patients who are undergoing dopamine replacement therapies. In our sample, 23.7% of PD patients exceeded the cut-off established in the literature for the ICD composite score (QUIP-ICD), whereas 26.3% of participants had at least one ICD domain that exceeded the cut-off threshold. This percentage aligns with findings reported in the literature (4, 5, 8, 11, 30, 38). Based on the results obtained, primary associations were consistently moderate-to-large in magnitude. A greater severity of ICD symptoms was associated with a more dysfunctional metacognitive profile. Specifically, higher ICD symptom severity scores were associated with positive beliefs about worry, negative beliefs about uncontrollability and danger, reduced cognitive confidence, greater need to control thoughts, and higher dysfunctional MBs in general. Conversely, higher ICD symptoms severity was negatively associated with cognitive self-consciousness. These associations remained significant even after accounting for dopamine agonist levodopa equivalent daily dose (LEDD-DA), non-dopamine agonist levodopa equivalent daily dose (nonDA-LEDD), and treatment duration. PD patients who met the QUIP-ICD cut-off were compared with those who did not; we observed that the most significant differences between groups were at the level of negative beliefs of uncontrollability and danger (MCQ-NEG), significantly higher in the QUIP ICD-positive group, and at the level of cognitive self-consciousness (MCQ-CSC), significantly lower in the QUIP ICD-positive group.

Regarding dopaminergic exposure variables, dopamine agonist exposure was positively associated with binge eating symptoms, while treatment duration was positively associated with dopamine dysregulation symptoms and overall QUIP-RS severity. In complementary analyses controlling for MCQ-TOT, the associations with binge eating and dopamine dysregulation symptoms remained significant, whereas the association between treatment duration and QUIP-RS total score was attenuated. This pattern suggests that adjustment for overall metacognitive beliefs had a differential influence on the observed associations. However, these findings should be interpreted cautiously in light of the limitations of this study.

The present findings can be situated within an emerging body of research extending the metacognitive framework to neurological populations. Maladaptive metacognitive beliefs have been associated with emotional distress and illness-related adjustment in Parkinson’s disease (39, 40), multiple sclerosis (41), epilepsy (42), amyotrophic lateral sclerosis (43), and stroke (44, 45). Although this literature has primarily focused on emotional distress rather than bahavioral dysregulation, the recurrence of these associations across neurologically distinct conditions supports the broader applicability of metacognitive theory to neurological populations. The present findings extend this literature by suggesting that, in PD, dysfunctional metacognitive beliefs may also be associated with individual differences in ICD symptom severity in the context of dopaminergic exposure.

Beyond neurological populations, dysfunctional metacognitive beliefs have been associated with a range of excessive and dysregulated bahaviors, including substance-related and bahavioral addictions (21, 46). From a broader theoretical perspective, this is consistent with the transdiagnostic nature of the S-REF/MCS framework, according to which maladaptive metacognitive beliefs and dysfunctional metacognitive control may contribute to difficulties in self-regulation across clinically distinct conditions (18, 47).

An addiction-specific triphasic formulation has also applied S-REF principles to the regulation of cognition before, during, and after engagement in addictive bahavior (20). This formulation provides a point of conceptual overlap with the present findings insofar as it proposes that metacognitive beliefs may be relevant to the regulation of cognition surrounding excessive bahavior. However, the relevance of this literature should not be taken to imply that ICDs and addictions of general populations share the same neurobiological mechanisms, or that the processes underlying substance-related addictions, bahavioral addictions, and ICDs are equivalent. Rather, consistent with the transdiagnostic premise of the S-REF/MCS model, similar forms of dysfunctional metacognitive beliefs may be relevant to maladaptive self-regulation across otherwise distinct clinical and neurobiological conditions.

In the context of PD, this framework may be particularly relevant. Contemporary models describe ICD symptoms as the result of an interaction between dopaminergic pharmacological stressors and interindividual susceptibility factors within mesocorticolimbic and frontostriatal systems. Within these models, dopaminergic stimulation may alter reward learning, incentive salience, and inhibitory control through mechanisms such as dopaminergic “overdose” of relatively preserved ventral striatal circuits and sensitization of reward-related pathways, with particular relevance of D2/D3 receptor-mediated mechanisms in the ventral striatum and nucleus accumbens (9, 48, 49). Neuroimaging and experimental studies have further reported greater ventral striatal dopamine release during gambling or reward-cue exposure, altered corticostriatal and intrinsic connectivity, reward hypersensitivity, and abnormal frontal value signaling in PD patients with ICDs symptoms (50–55). Our data do not allow any direct inference about these neural mechanisms, because the study did not include neuroimaging, receptor availability indices, experimental reward-processing tasks, or longitudinal medication trajectories. Nevertheless, the results allow a hypothesis-generating psychological interpretation. MBs should not be viewed as an alternative explanation to dopaminergic or frontostriatal models. Rather, they may represent one possible cognitive-affective correlate through which patients appraise and regulate internal experiences, such as urges, intrusive images, reward-related thoughts, craving-like states, or distress, within a neuropharmacological context that may already increase reward salience.

Several limitations should be acknowledged. First of all, the sample size was small. While FDR correction, confidence intervals, and *post hoc* sensitivity power analysis improve the statistical reporting, they do not remove the risk of overestimating some associations. Second, the cross-sectional design prevents any conclusion about temporality, directionality, or causality. Dysfunctional metacognitive beliefs may contribute to the maintenance or amplification of ICD symptoms, but recurrent ICD behaviors may also worsen metacognitive beliefs, increase perceived uncontrollability, reduce cognitive confidence, or alter self-monitoring. A bidirectional relationship is therefore plausible; only longitudinal research will be able to clarify the specific direction and confirm or refute our hypothesis. Third, the available pharmacological indices only partially capture cumulative dopaminergic exposure. The analyses do not capture previous maximum dose, medication changes over time, or the temporal relationship between dopaminergic treatment and ICD onset. Fourth, all participants were recruited from an inpatient rehabilitation setting. This setting allowed detailed multidisciplinary clinical assessment, but it may also introduce selection bias. Inpatients may differ from outpatient or community PD populations in disease burden, rehabilitation needs, cognitive profile, psychiatric comorbidity, medication complexity, or caregiver involvement. Accordingly, the present findings should not be generalized uncritically. Finally, although psychotropic medication exposure and available dose-burden indices were not associated with ICD status or QUIP-ICD severity, these variables should be considered only indirect proxies of psychiatric burden, therefore, residual neuropsychiatric confounding cannot be ruled out.

In conclusion, this study provides the first exploratory evidence of the association between ICD symptoms and dysfunctional MBs in patients with PD undergoing dopamine replacement therapies. Future longitudinal studies should examine whether metacognitive characteristics are prospectively associated with the onset, persistence, or progression of ICD symptoms, beyond the contribution of pharmacological and clinical factors. Such studies could clarify whether metacognitive characteristics may have potential utility in the screening, monitoring, and integrated care of patients with PD, with possible implications for the well-being of patients and their caregivers.

## Data Availability

The raw data supporting the conclusions of this article will be made available by the authors, without undue reservation.
