# Supplementary material for: The role of metacognitive beliefs in impulse control disorders and addictive-like behaviors in Parkinson’s disease: an exploratory study
Source: Front Psychiatry. 2026 Sep 3;17:1893510. doi: 10.3389/fpsyt.2026.1893510 (PMC13582548; doi:10.3389/fpsyt.2026.1893510)
Supplement: Supplementary file 1 [file DataSheet1.pdf]

# *Supplementary Material*

## 1 Supplementary Tables

**Table S1.** Correlations between MCQ-30 dimensions and ICD composite outcomes controlled for dopaminergic exposure and treatment duration

| Outcome | MCQ-30 domain | Partial r | 95% CI | p | pFDR |
|---|---|---|---|---|---|
| QUIP-ICD (gambling+Hypersexuality+Compulsive Shopping+Binge Eating) | MCQ-POS | .511 | [.249, .768] | .002 | .002 |
| | MCQ-NEG | .606 | [.433, .792] | < .001 | .001 |
| | MCQ-CC | .481 | [.202, .748] | .003 | .003 |
| | MCQ-NC | .559 | [.298, .794] | < .001 | .001 |
| | MCQ-CSC | -.623 | [-.791, -.470] | < .001 | .001 |
| | MCQ-TOT | .547 | [.292, .785] | < .001 | .001 |
| QUIP-RS total (QUIP-ICD+Hobby&Punding+Dopamine Dysregulation Syndrome) | MCQ-POS | .473 | [.200, .728] | .004 | .004 |
| | MCQ-NEG | .705 | [.542, .868] | < .001 | .001 |
| | MCQ-CC | .630 | [.375, .874] | < .001 | .001 |
| | MCQ-NC | .586 | [.332, .810] | < .001 | .001 |
| | MCQ-CSC | -.702 | [-.845, -.568] | < .001 | .001 |
| | MCQ-TOT | .629 | [.399, .838] | < .001 | .001 |

*Note. Partial correlations controlled for dopamine agonist exposure (LEDD-DA), non-dopamine agonist dopaminergic exposure (nonDA-LEDD), and treatment duration. N = 38; df = 33. 95% confidence intervals were estimated using bootstrap procedures. pFDR values were adjusted using the Benjamini-Hochberg false discovery rate procedure within the primary family of tests (12 tests). For p values reported as < .001, p = .001 was used for FDR computation.*

**Table S2.** Exploratory domain-level correlations between QUIP-RS domains and MCQ-30 dimensions controlled for dopaminergic exposure and treatment duration

| Outcome | MCQ-30 domain | Partial r | 95% CI | p | pFDR |
|---|---|---|---|---|---|
| Gambling | MCQ-POS | .283 | [.011, .521] | .100 | .120 |
| | MCQ-NEG | .391 | [.167, .612] | .020 | .045 |
| | MCQ-CC | .336 | [.117, .585] | .048 | .079 |
| | MCQ-NC | .412 | [.122, .650] | .014 | .042 |
| | MCQ-CSC | -.371 | [-.569, -.177] | .028 | .053 |
| | MCQ-TOT | .395 | [.131, .628] | .019 | .045 |
| Hypersexuality | MCQ-POS | .390 | [.058, .594] | .020 | .045 |
| | MCQ-NEG | .509 | [.284, .727] | .002 | .012 |
| | MCQ-CC | .380 | [.037, .665] | .024 | .048 |
| | MCQ-NC | .434 | [.108, .709] | .009 | .029 |
| | MCQ-CSC | -.487 | [-.706, -.278] | .003 | .015 |
| | MCQ-TOT | .446 | [.123, .702] | .007 | .028 |
| Compulsive shopping | MCQ-POS | .381 | [.047, .626] | .024 | .048 |
| | MCQ-NEG | .367 | [.083, .577] | .030 | .054 |
| | MCQ-CC | .181 | [-.256, .519] | .297 | .314 |
| | MCQ-NC | .331 | [.000, .619] | .052 | .081 |
| | MCQ-CSC | -.219 | [-.411, -.028] | .207 | .233 |
| | MCQ-TOT | .402 | [-.019, .669] | .017 | .045 |
| Binge eating | MCQ-POS | .290 | [-.035, .595] | .091 | .113 |
| | MCQ-NEG | .309 | [.046, .553] | .071 | .104 |
| | MCQ-CC | .337 | [.028, .618] | .048 | .079 |
| | MCQ-NC | .295 | [.024, .538] | .085 | .109 |

| | | | | | |
|---|---|---|---|---|---|
| | MCQ-CSC | -.504 | [-.690, -.257] | .002 | .012 |
| | MCQ-TOT | .212 | [-.053, .477] | .221 | .241 |
| Hobby & punding | MCQ-POS | .306 | [.016, .592] | .074 | .104 |
| | MCQ-NEG | .554 | [.329, .772] | < .001 | .012 |
| | MCQ-CC | .500 | [.250, .724] | .002 | .012 |
| | MCQ-NC | .477 | [.252, .724] | .004 | .018 |
| | MCQ-CSC | -.523 | [-.725, -.343] | .001 | .012 |
| | MCQ-TOT | .505 | [.259, .746] | .002 | .012 |
| Dopamine dysregulation syndrome | MCQ-POS | .019 | [-.342, .307] | .916 | .916 |
| | MCQ-NEG | .305 | [.060, .533] | .075 | .104 |
| | MCQ-CC | .441 | [.186, .627] | .008 | .029 |
| | MCQ-NC | .058 | [-.203, .395] | .740 | .761 |
| | MCQ-CSC | -.301 | [-.525, -.033] | .079 | .105 |
| | MCQ-TOT | .236 | [.033, .457] | .173 | .201 |

*Note. Domain-level analyses are exploratory. Partial correlations controlled for LEDD-DA, nonDA-LEDD, and treatment duration. N = 38; df = 33. 95% confidence intervals were estimated using bootstrap procedures. pFDR values were adjusted using the Benjamini-Hochberg procedure within the secondary domain-level family of tests (36 tests). For p values reported as < .001, p = .001 was used for FDR computation.*

**Table S3. Exploratory group comparisons of MCQ-30 scores between ICD-positive and ICD-negative patients**

| MCQ-30 domain | ICD-positive M ± SD | ICD-negative M ± SD | Mean difference | 95% CI for mean difference | t(df) | p | pFDR | Cohen's d | 95% CI for Cohen's d |
|---|---|---|---|---|---|---|---|---|---|
| MCQ-POS | 11.22 ± 1.72 | 8.86 ± 2.67 | 2.36 | [0.79, 3.93] | 3.12 (21.12) | .005 | .010 | 0.95 | [0.16, 1.72] |
| MCQ-NEG | 20.89 ± 2.57 | 15.72 ± 5.19 | 5.17 | [2.52, 7.81] | 4.01 (28.15) | < .001 | .003 | 1.09 | [0.30, 1.87] |
| MCQ-CC | 17.33 ± 5.68 | 14.55 ± 4.80 | 2.78 | [-1.09, 6.66] | 1.46 (36) | .154 | .154 | 0.56 | [-0.21, 1.31] |
| MCQ-NC | 17.22 ± 3.07 | 14.03 ± 3.35 | 3.19 | [0.64, 5.74] | 2.54 (36) | .016 | .024 | 0.97 | [0.18, 1.74] |
| MCQ-CSC | 9.22 ± 3.15 | 16.00 ± 6.19 | -6.78 | [-9.97, -3.59] | -4.35 (27.37) | < .001 | .003 | -1.20 | [-1.99, -0.39] |
| MCQ-TOT | 75.89 ± 7.57 | 69.17 ± 8.94 | 6.72 | [0.02, 13.41] | 2.03 (36) | .049 | .059 | 0.78 | [0.00, 1.54] |

*Note. ICD-positive indicates patients exceeding the QUIP-ICD cut-off; ICD-negative indicates patients below the cut-off. Group differences are computed as ICD-positive minus ICD-negative. Welch t-tests are reported for MCQ-POS, MCQ-NEG, and MCQ-CSC; Student independent-samples t-tests are reported for MCQ-CC, MCQ-NC, and MCQ-TOT, consistently with the analyses reported in the main text. 95% CIs for mean differences correspond to the reported t-test rows. pFDR values were adjusted using the Benjamini-Hochberg false discovery rate procedure across the six MCQ-30 group comparisons. Cohen's d is based on the pooled standard deviation. N = 38; ICD-positive n = 9; ICD-negative n = 29.*
